# The influence of a semi-arid sub-catchment on suspended sediments in the Mara River, Kenya

**DOI:** 10.1371/journal.pone.0192828

**Published:** 2018-02-08

**Authors:** Christopher L. Dutton, Amanda L. Subalusky, Shimon C. Anisfeld, Laban Njoroge, Emma J. Rosi, David M. Post

**Affiliations:** 1 Department of Ecology and Evolutionary Biology, Yale University, New Haven, CT, United States of America; 2 Cary Institute of Ecosystem Studies, Millbrook, New York, United States of America; 3 School of Forestry and Environmental Studies, Yale University, New Haven, CT, United States of America; 4 Invertebrate Zoology Section, The National Museums of Kenya, Nairobi, Kenya; Centre National de la Recherche Scientifique, FRANCE

## Abstract

The Mara River Basin in East Africa is a trans-boundary basin of international significance experiencing excessive levels of sediment loads. Sediment levels in this river are extremely high (turbidities as high as 6,000 NTU) and appear to be increasing over time. Large wildlife populations, unregulated livestock grazing, and agricultural land conversion are all potential factors increasing sediment loads in the semi-arid portion of the basin. The basin is well-known for its annual wildebeest (*Connochaetes taurinus*) migration of approximately 1.3 million individuals, but it also has a growing population of hippopotami (*Hippopotamus amphibius*), which reside within the river and may contribute to the flux of suspended sediments. We used *in situ* pressure transducers and turbidity sensors to quantify the sediment flux at two sites for the Mara River and investigate the origin of riverine suspended sediment. We found that the combined Middle Mara—Talek catchment, a relatively flat but semi-arid region with large populations of wildlife and domestic cattle, is responsible for 2/3 of the sediment flux. The sediment yield from the combined Middle Mara–Talek catchment is approximately the same as the headwaters, despite receiving less rainfall. There was high monthly variability in suspended sediment fluxes. Although hippopotamus pools are not a major source of suspended sediments under baseflow, they do contribute to short-term variability in suspended sediments. This research identified sources of suspended sediments in the Mara River and important regions of the catchment to target for conservation, and suggests hippopotami may influence riverine sediment dynamics.

## Introduction

Excessive sediment loads in rivers are a concern throughout the world as suspended sediments can be a major transport medium for chemicals, contaminants and nutrients [1–3]. Human and aquatic systems are directly at risk, as downstream impacts of suspended sediment transport on water quality and biota can be significant [4–7]. In instances of trans-boundary rivers, excessive sediment loads across international borders could lead to destabilized political relations and ultimately conflict [8]. A clear understanding of the major sources of suspended sediments is required in order to manage the problem [9].

Suspended sediments are primarily a mix of organic and inorganic terrestrially derived particles [10]. Turbidity can be correlated with suspended sediment concentration and then used as a surrogate, which allows for high resolution data on suspended sediment concentrations to be estimated for remote sites [11–13]. These data are necessary for accurately calculating sediment flux and yields in flashy rivers [14], which is an important step in understanding and mitigating the sources of suspended sediments. Suspended sediment flux data has been important for understanding a variety of rivers [9, 15], but there is a paucity of suspended sediment flux data for African rivers [3, 16].

The Mara River, which is shared by Kenya and Tanzania, is a trans-boundary river of tremendous conservation importance, and sedimentation and non-point source pollution have become issues of major concern in recent years [17]. The Mara River provides the only permanent source of water for two of the most famous protected areas in the world–the Maasai Mara National Reserve (MMNR) in Kenya and the Serengeti National Park in Tanzania. Despite portions of the river being surrounded by protected areas, the river basin has undergone major changes in land use over the last 50 years and those changes have influenced water quantity and quality in the river [18–20]. Increased sediment loads in the river have been blamed for increased pollutant loads [21] and a 4-fold increase in the size of the Mara Wetland near the mouth of the river [18]. Management authorities have found that excessive levels of total suspended solids (TSS) and nutrients in the Mara River may be playing a role in the eutrophication of Lake Victoria [22–24]. The waters of the Mara also sustain nearly one million people, many of whom are rural poor, with 62% directly reliant upon the river for their domestic water needs [25–27].

The majority of studies on the Mara River have focused on soil erosion in the upper catchment of the river basin as the source for increasing sediment loads in the river [19, 28–30]. The upper catchment of the Mara is currently under threat from illegal logging and encroachment from a rapidly growing population [31]. Studies conducted in the upper catchment of the Mara River have found excessive levels of suspended sediments contributing to an overall decline in the health of the aquatic ecosystem [27–29, 32]. Other studies have linked deforestation in the Mau Forest, which forms the headwaters of the Mara, to changes in the hydrology of the system [18, 20]. Few studies have examined the contribution of other regions of the catchment to sediment levels in the Mara, despite concerns about rapid development of tourism establishments in the middle portions of the catchment, along with substantial grazing pressure by domestic livestock [33, 34]. We used sediment fingerprinting to quantify the sources of suspended sediment in the Mara River during a three month period in 2011, and results from that study suggested that a semi-arid sub-catchment of the river may be generating up to 2/3 of the suspended sediments in the river [35].

One potentially important, but under-studied, driver of sediment generation processes is large wildlife. There are large numbers of a diversity of wildlife species that use the Mara River, including 1.3 million wildebeest during their annual migration from the Serengeti, in addition to large numbers of livestock, all of which may influence sediment generation and flux in the river. Here, we focus on the influence of hippopotami (*Hippopotamus amphibius*, hereafter referred to as hippos), due to their high density in the Mara and their direct influence on in-stream processes. Hippos are known as ecosystem engineers, capable of exerting a transformative effect upon their environment [36, 37]. Hippos graze in terrestrial systems at night, and spend the day wallowing in aquatic systems, and their movements within and between these systems can alter the geomorphology, nutrient cycling and species composition of both ecosystems [38, 39]. The stirring effects of hippo movements within hippo pools can aerate and vertically mix the water column while disturbing organic and inorganic sediments on the bottom [40]. Prior results from a sediment fingerprinting study in the Mara showed that hippo feces could account for 5% of suspended sediments in the Mara [35]. Thus, hippos may influence both the generation of organic sediment (through defecation) and the remobilization of inorganic and organic sediments within the river channel. These processes may be particularly important in the Mara River, which has a large population of over 4,100 hippos at a density of 27 hippos per river kilometer, loading >32,000 kg of feces daily [39, 41]. These processes also may be relevant for understanding past sediment dynamics in other sub-Saharan rivers where hippo populations are declining or gone [42].

In this study, we used *in situ* pressure transducers and turbidity loggers to quantify the sediment flux at two sites for the Mara River over a period of 3 years, and we separated the contribution of the upper, forested catchment from the main catchment in the middle Mara and a semi-arid catchment that drains the seasonal Talek River. We also used focal measurements of hippo pools to measure the effect that hippos have on turbidity in the Mara River.

## Methods

### Study area

**Fig 1:**
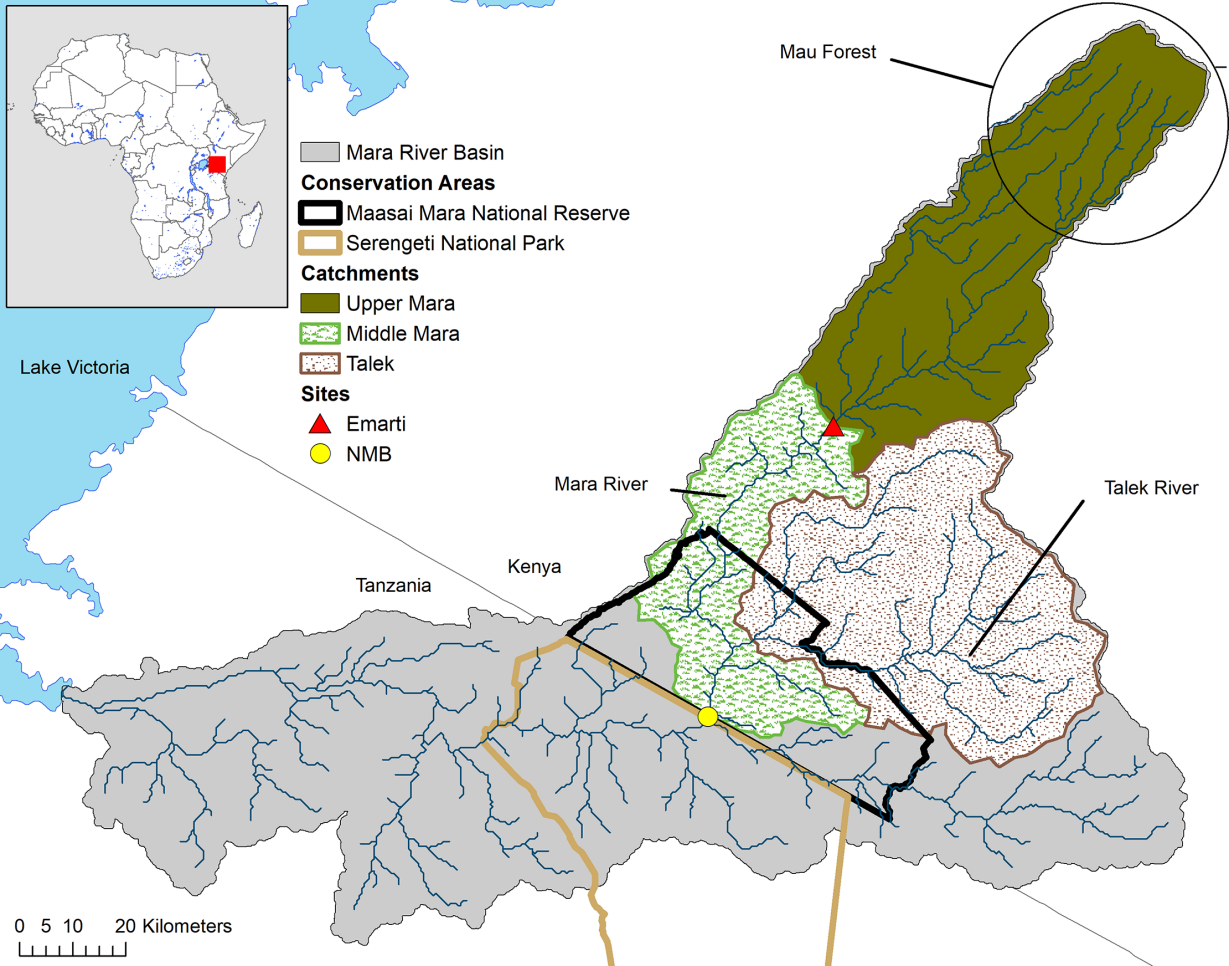
Map of the Mara River Basin, catchments and sites.

The Mara River is a trans-boundary waterway shared between Kenya and Tanzania (Fig 1). The upper catchment of the basin is a remnant of the largest indigenous montane forest in East Africa, and receives approximately 1400 mm rainfall per year [43, 44]. The eastern side of the basin receives approximately 600 mm rainfall per year and is drained by the Talek River, a seasonal river that floods in response to isolated showers within the Olare Orok, Ntiantiak, Sekanani and Loita drainages [18]. The Talek River joins the Mara River approximately 16 kilometers north of the border between Kenya and Tanzania. Discharge patterns in the Mara River Basin are bimodal, reflecting the occurrence of a short and long rainy season [45]. In the upper Mara (Emarti), discharge peaks in May and August-September, and in the lower Mara (NMB), discharge peaks in April-May and December, with contributions from seasonal rains in the Middle Mara and Talek catchments [45].

Two primary sites were utilized to separate the study area into two distinct catchment areas: Upper Mara and the combined catchments of Middle Mara and Talek (Fig 1). The most upstream monitoring site is Emarti (36M 748332E, 9883150S), which is located at Emarti Bridge, just downstream of the confluence of the Mara’s two primary tributaries, the Amala and Nyangores Rivers. Emarti integrates the influence of the river’s mountainous headwaters and small-scale farming and urban development. This site is upstream of the majority of large wildlife and wildlife conservancies.

The New Mara Bridge site (NMB) (36M 724636E 9828995S) is located at a bridge just upstream of the border between Kenya and Tanzania. This site integrates the influence of Emarti (38% of the NMB catchment), the Talek (41% of the NMB catchment) and the Middle Mara region (21% of the NMB catchment). The Middle Mara region has no other perennial rivers contributing to the Mara. Land use in the Middle Mara region is primarily pastoralism, tourism developments and wildlife conservancies. Approximately half of the Middle Mara region is within the MMNR.

### Hydrology

At Emarti, we measured stage height every 15 minutes from June 2011 –Dec 2014 using a RuggedTroll 100 pressure transducer (In-Situ Inc., Fort Collins, CO, USA). At NMB, stage height was measured every 15 minutes from June 2011 –November 2012 using a RuggedTroll 100 pressure transducer and from December 2012 –December 2014 using a pressure transducer connected to a Eureka Manta2 sonde (Eureka Water Probes, Austin, TX, USA). All measurements from the pressure transducers were corrected for atmospheric pressure changes with a BaroTroll that was also installed at each site (In-Situ Inc., Fort Collins, CO, USA). From April–August 2014, the sonde at the NMB was not operational, so stage height was measured every 30 minutes using an Arduino-based ultrasonic sensor (Maxbotix Inc., Brainerd, MN, USA).

To develop rating curves for stage-discharge relationships at the two sites, we measured discharge on multiple occasions in 2011 (4 at Emarti, 10 at NMB) with the area-velocity method using a handheld staff gauge or weighted measuring tape for depth and velocimeter for velocity. In 2014, we measured discharge 3 times at Emarti and once at NMB using either the measuring tape and velocimeter or a HydroSurveyor (SonTek, San Diego, CA, USA). Bedrock substrate is present in both channel reaches where rating curves were developed, so we assumed channel geomorphology was consistent over time.

**Fig 2:**
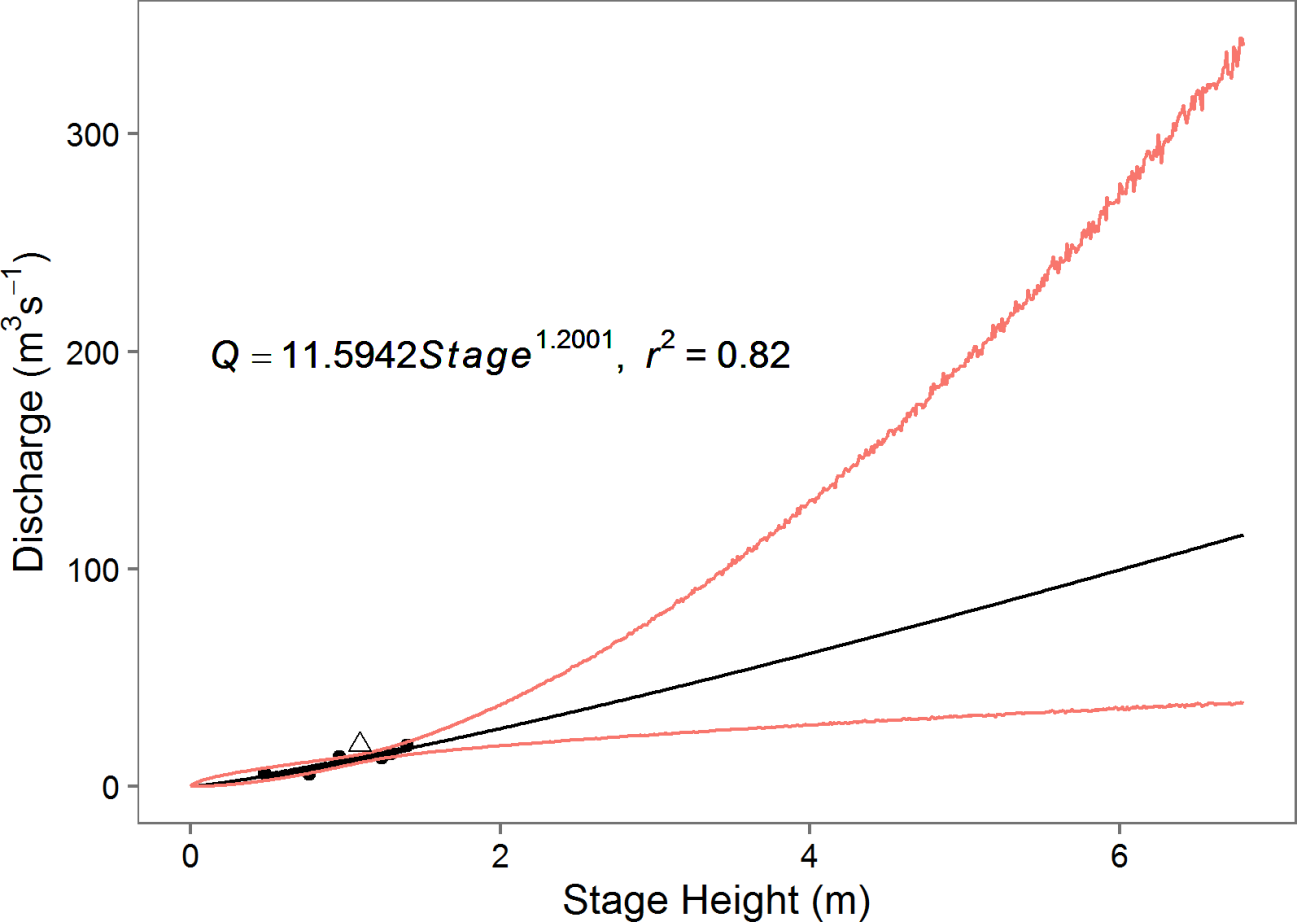
Stage height to discharge rating curves for Emarti extrapolated over the full range of measured stage height. Outliers that were removed are presented as a triangle. 95% confidence intervals are in red.

**Fig 3:**
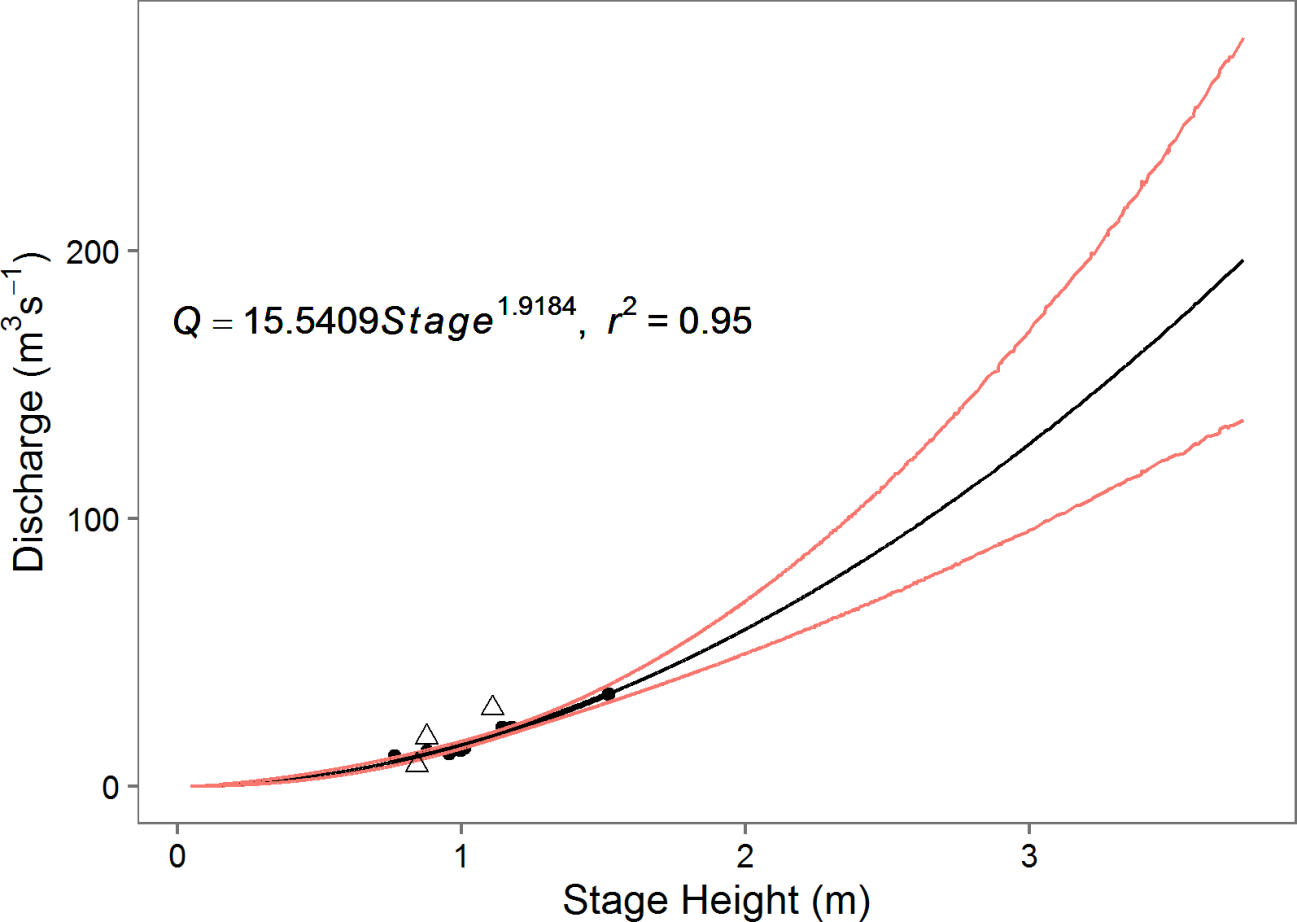
Stage height to discharge rating curves for December 2012 –December 2014 at NMB extrapolated over the full range of measured stage height. Outliers that were removed are presented as a triangle. 95% confidence intervals are in red.

We converted stage height to discharge using a rating curve developed for each site in R with the nls function [46]. The rating curve developed for Emarti has a R^2^ of 0.82 (Fig 2). Two curves were developed for the NMB site because the pressure transducer in the sonde installed in December 2012 was installed 0.25m deeper than the earlier installation (June 2011 –Nov 2012). Because both installations were at the same horizontal profile, all discharge measurements taken between 2011 and 2014 were used in each rating curve. Both rating curves have a R^2^ of 0.95 (Fig 3 and S1 Fig). Outliers were removed from the rating curves if they were collected at non-optimal flow conditions (e.g. rising or falling flood pulse) and fell outside of the initial 95% confidence intervals. We removed one measurement from the 2014 Emarti dataset, and three from the NMB dataset (shown as triangles in Figs 2 and 3 and S1 Fig). A Monte Carlo simulation was utilized to extend the regression through all observed stage height values and to generate 95% confidence intervals based on the propagation of errors from our model predictor variables and the fit parameters [47]. Monthly mean, coefficient of variation (CV), minimum and maximum discharges were calculated for each site.

The Richards-Baker Flashiness Index (R-B Index) was calculated for Emarti and NMB using mean daily discharge data over each year [48]. The index is calculated by summing the absolute value of change in flow over two consecutive days for the entire year then dividing by the sum of daily flows for that year. The R-B Index provides a value indicating the “flashiness” of a river that can be used for comparing between years or between sites. Higher R-B Index values indicate a “flashier” river. The R-B Index is commonly used to quantify the frequency and magnitude of short-term changes in river flow.

### Sediment fluxes

We measured *in situ* turbidity at the Emarti and NMB sites using a NEP9500 turbidity sensor (McVan Instruments Pty Ltd., Mulgrave, Australia) built into a Manta2 water quality data logger (Eureka Water Probes, Austin, TX, USA). Manta2 water quality data loggers were installed at the Emarti and NMB sites intermittently from June 2011 through December 2014. The sondes were programmed to log every 15 minutes. Prior to taking a measurement, the sonde would wipe the turbidity probe to prevent fouling. Sondes were calibrated according to manufacturer instructions. The turbidity sensor is certified up to 3000 NTU, however, it is capable of providing a reading over 4000 NTU. During several floods, turbidity values exceeded what was readable by the sensor at both sites.

We collected 44 water samples at both sites (16 Emarti, 28 at NMB) during dry and wet seasons to calculate suspended sediment concentrations relative to turbidity. In 2011, water samples were collected with a US DH-59 depth integrating suspended sediment sampler suspended from bridges at Emarti and NMB. Samples were taken from the most representative and accessible cross section of each site. Care was taken to ensure an equal transit time throughout the depth of the vertical depth profile. From 2012–2014, water samples were taken with a 1-liter Nalgene bottle from a well-mixed representative reach at each site. All water samples were taken in accordance with USGS methods [49].

We filtered a known volume of water through a pre-weighed cellulose nitrate (CN) filter (Whatman, 47mm, 0.45μm pore size) immediately after sample collection. We dried CN filters with suspended sediment in a solar oven immediately in the field and sealed the samples for transport to the US. Upon arrival in the US, the filter papers were dried in an oven at 60°C for 24h and re-weighed to calculate the mass of the sediment.

**Fig 4:**
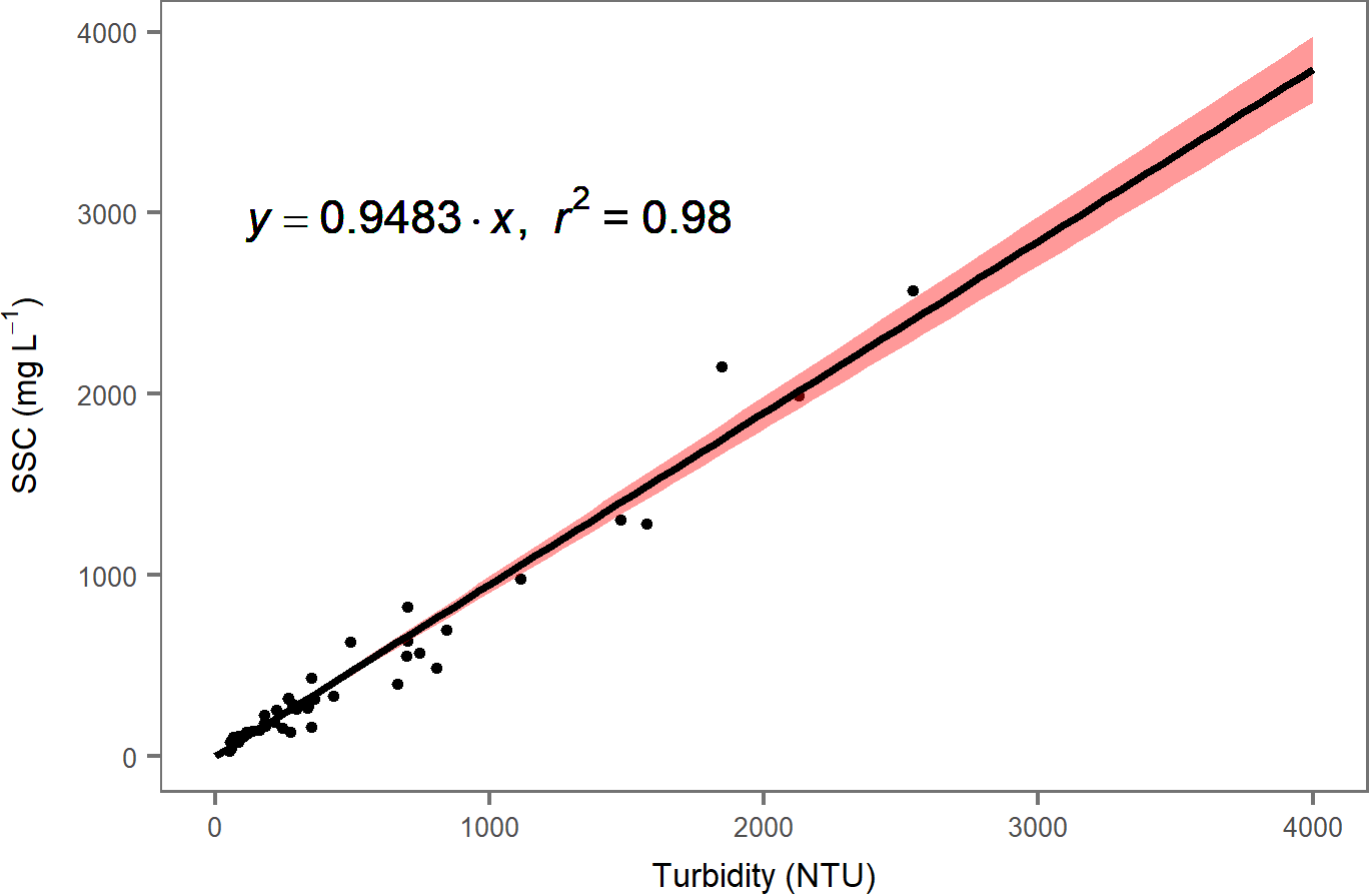
Turbidity to suspended sediment concentration rating curve for Emarti and NMB. 95% confidence interval is shaded in red.

We used a simple linear regression to relate instantaneous Manta2 turbidity data (NTU) to suspended sediment concentration (mg L^-1^) for Emarti and NMB sites with the lm function in R [46]. 95% confidence intervals were generated with the predict function in R [46]. The regression converting SSC to turbidity has a factor of 0.9483 and a R^2^ of 0.98 (Fig 4). We calculated sediment flux (in tonne day^-1^) by multiplying instantaneous estimated river discharge (m^3^ s^-1^) by the instantaneous estimated suspended sediment concentration (mg L^-1^) obtained from our SSC:turbidity regression. We then calculated the mean per month and mean over the entire period of record. We did not calculate total annual flux, as we were missing months for each year of record. Uncertainty in flux was calculated with the sum in quadrature of the fractional uncertainties in the stage height to discharge rating curve and the turbidity to SSC regression [50].

We estimated catchment area of each sampling location using drainage patterns derived from the 15m ASTER Global DEM dataset, Version 2. Analysis was done with ArcGIS 10 using the Spatial Analyst toolbox and the Arc Hydro extension [51]. Sediment yield (tonnes km^-2^ year^-1^) was computed by dividing the average instantaneous suspended sediment flux over the record by the catchment area (km^2^). Yield was also computed for the combined Middle Mara and Talek regions by subtracting the Emarti flux from the NMB flux and dividing by the catchment area of the combined Middle Mara and Talek regions. We did not calculate total annual yield, as we were missing months for each year of record.

The focus of this analysis was to compare sediment fluxes between Emarti and NMB, so we restricted analysis to dates when we had data for both sites. Travel time between those two sites is highly variable in this system and is dependent on discharge, thus we did not calculate a general time offset for data between Emarti and NMB. However, travel time between the sites is typically within 1–2 days, which likely had minimal effect on our analysis of monthly comparisons.

To aid in visualization, data were divided into 8 blocks of continuous coverage with no data gap greater than one week (S1 Table). The time blocks vary in size from 14 to 95 days. The number of overlapping measurements taken from both sites was utilized to generate a coverage percentage per month and year between 2011 and 2014. Total monthly coverage of overlapping data varied between 4% in December 2012 to 99% in July 2011 (S2 Table).

### Sediment dynamics of hippo pools

We measured turbidity upstream and downstream of four different hippo pools for 24–48 hours (S3 Table) in order to understand the dynamics of hippos on sediment transport. Each pool was measured once. A Manta2 water quality sonde was placed into the river upstream of a hippo pool and a second sonde was placed just downstream of the hippo pool. The hippos in each pool were counted.

Turbidity from the hippo pools were not normally distributed, and data points within each hippo pool were not independent from one another, so we used non-parametric tests that accounted for sample dependence. Because we were examining fine-scale patterns in suspended sediment concentrations, we accounted for travel time between the upstream and downstream measurements by the sondes. We estimated for each time-series the time lag in one minute increments (between 0 and 18 minutes) as the lag that maximized the Kendall Rank Correlation Coefficient (tau) in turbidity between upstream and downstream time-series [46]. The coefficient of variation was calculated for each set of turbidity measurements by dividing the standard deviation by the mean. A non-parametric Wilcoxon Signed Rank test was used to compare upstream and downstream means from each hippo pool [46]. Levene’s test was used to compare the upstream and downstream variances from each hippo pool [52]. Differences between upstream and downstream values were considered significant with an α value of 0.05 for both tests.

### Ethics statement

This study was authorized by the Government of Kenya and the National Council for Science and Technology (Research permits NCST/RRI/12/1/BS-011/25 and NCST/RDB/12B/012/36). Permission to conduct this research within the Masai Mara National Reserve was granted by the Transmara and Narok County Councils.

## Results

### Hydrology

**Fig 5:**
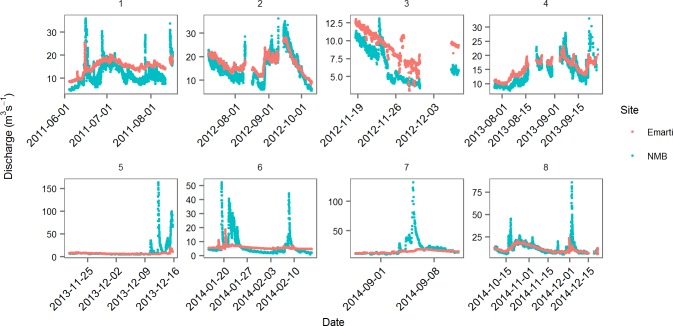
Hydrograph for Emarti and NMB during/across eight time blocks from 2011 to 2014.

The hydrographs for the two sites show that the Emarti site has the highest baseflow discharge and that there is often water loss between the Emarti and NMB sites during baseflow conditions (Fig 5). Average discharge over this period of measurement was not statistically different between the two sites, although variability was much higher at NMB (higher CV). Average discharge for the Emarti site during this study was 13.3 m^3^ s^-1^ (_- 23%_^+ 32%^, _lower 95% CI_^upper 95% CI^) and average discharge from NMB was 12.5 m^3^ s^-1^ (_- 15%_^+ 19%^). The spikes in discharge from the NMB site that were not present at the Emarti site are largely due to the influence of the Talek River, as there are no other rivers entering the Mara within this reach, although overland flow and seasonal drainages could also be a factor (Fig 5).

Monthly mean discharge was highest at Emarti in 14 of 21 months although maximum discharge never exceeded 30 m^3^ s^-1^ (Table 1). Maximum discharge at NMB regularly exceeded 30 m^3^ s^-1^ and the coefficient of variation was highest at NMB in 17 of the 21 months. Minimum discharge was consistently lower at NMB than Emarti except in two months (November 2012 and December 2014).

**Table 1:** Monthly mean discharge (m^3^ s^-1^), lower and upper 95% confidence intervals (CI), minimum discharge, maximum discharge and coefficient of variation (CV) for Emarti and NMB.

Month Year	Emarti						NMB					
	Mean	95% CI		Min	Max	CV	Mean	95% CI		Min	Max	CV
		Lower	Upper					Lower	Upper			
June 2011	13.4	19%	21%	8.3	25.6	0.3	10.8	16%	19%	4.6	36.0	0.5
July 2011	16.0	15%	16%	13.0	20.4	0.1	12.3	13%	14%	7.1	28.9	0.3
August 2011	15.8	15%	16%	13.9	20.2	0.1	12.6	13%	14%	7.5	33.9	0.4
July 2012	17.6	18%	20%	13.0	22.1	0.1	15.1	10%	11%	9.1	22.9	0.2
August 2012	15.3	16%	17%	11.5	23.9	0.2	12.0	14%	16%	6.1	28.6	0.3
September 2012	20.0	21%	26%	13.8	28.4	0.2	21.6	8%	8%	11.3	36.3	0.2
October 2012	11.2	19%	20%	8.2	14.0	0.1	9.3	16%	20%	5.6	15.6	0.3
November 2012	9.2	27%	36%	3.0	13.0	0.2	6.9	21%	26%	3.4	13.1	0.4
December 2012	9.4	24%	28%	8.3	9.9	0.0	5.9	20%	25%	5.2	6.6	0.1
July 2013	10.5	20%	22%	9.9	11.5	0.0	9.5	15%	16%	8.5	10.9	0.0
August 2013	14.5	17%	18%	9.8	20.1	0.2	12.1	12%	13%	7.5	23.1	0.3
September 2013	17.0	17%	19%	13.0	23.3	0.1	16.6	9%	9%	9.9	33.1	0.2
November 2013	7.7	31%	41%	5.9	9.6	0.1	7.3	18%	21%	5.4	9.5	0.1
December 2013	6.9	35%	52%	5.6	20.4	0.3	14.4	19%	22%	4.9	163.5	1.5
January 2014	6.3	38%	58%	4.8	18.7	0.2	8.2	22%	29%	2.0	52.3	1.0
February 2014	5.2	42%	70%	4.7	7.4	0.1	4.5	27%	38%	0.8	44.4	1.1
August 2014	11.9	17%	17%	11.0	13.0	0.0	11.3	12%	13%	9.3	13.4	0.1
September 2014	15.3	15%	16%	11.3	18.6	0.1	22.5	10%	11%	9.6	132.7	0.7
October 2014	14.1	19%	21%	8.7	21.1	0.3	14.8	12%	13%	6.1	45.6	0.4
November 2014	10.5	23%	28%	7.2	23.8	0.3	10.5	14%	16%	5.4	24.7	0.3
December 2014	9.2	26%	32%	6.0	15.1	0.2	13.2	15%	17%	6.2	86.1	0.9

NMB had a Richards-Baker Flashiness Index (R-B Index) score that was on average 3.5 times higher than that Emarti and that exceeded the R-B Index at Emarti in every year (Table 2). The R-B Index score for NMB increased in every year of this study from 0.131 in 2011 to 0.261 in 2014. The R-B Index also increased slightly for Emarti (0.044 in 2011 to 0.058 in 2014).

**Table 2:** Richards-Baker Flashiness Index.

Year	Richards-Baker Flashiness Index	
	NMB	Emarti
2011	0.131	0.044
2012	0.133	0.052
2013	0.222	0.061
2014	0.261	0.058

### Sediment fluxes

**Fig 6:**
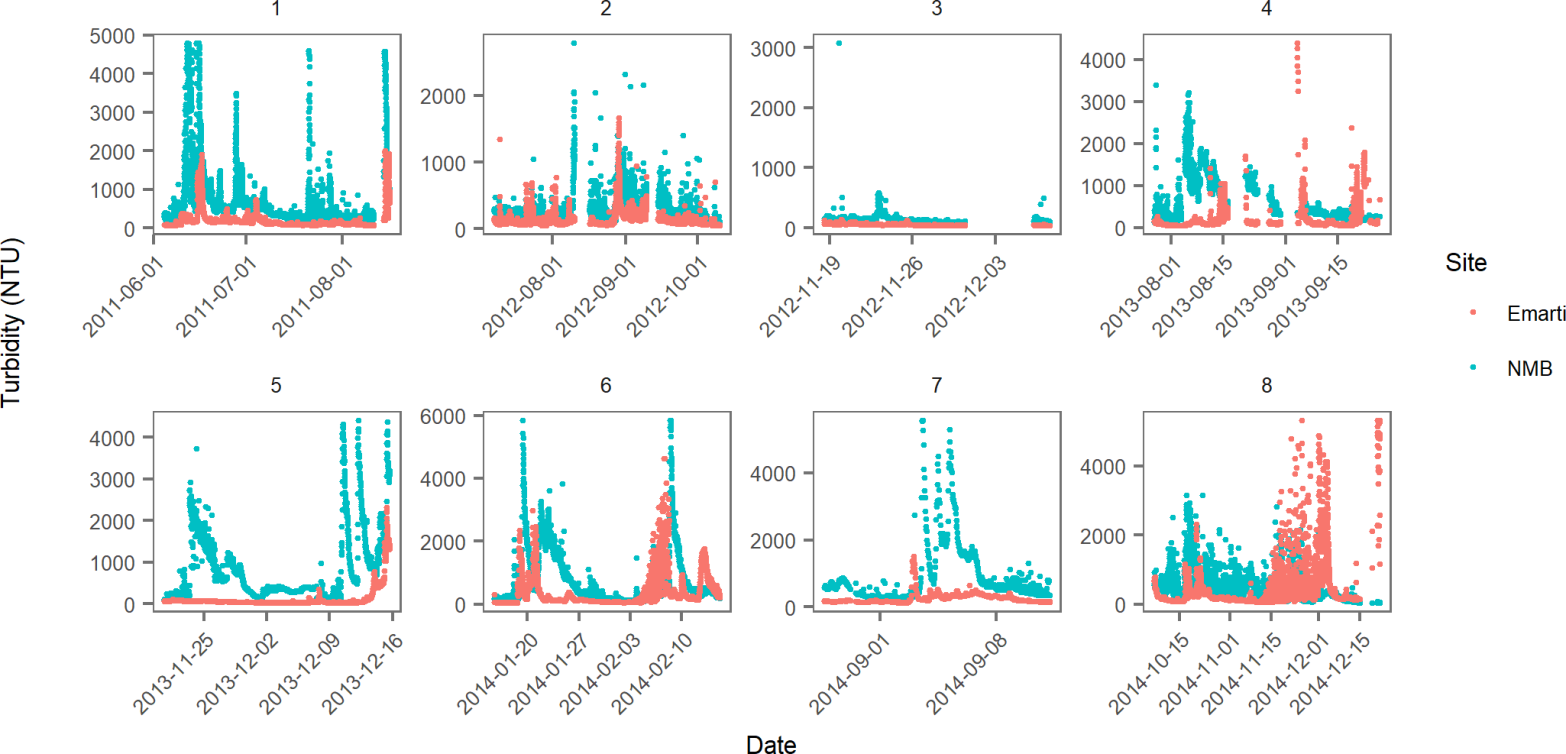
Turbidity from Emarti and NMB during the eight time blocks from 2001 to 2014.

**Fig 7:**
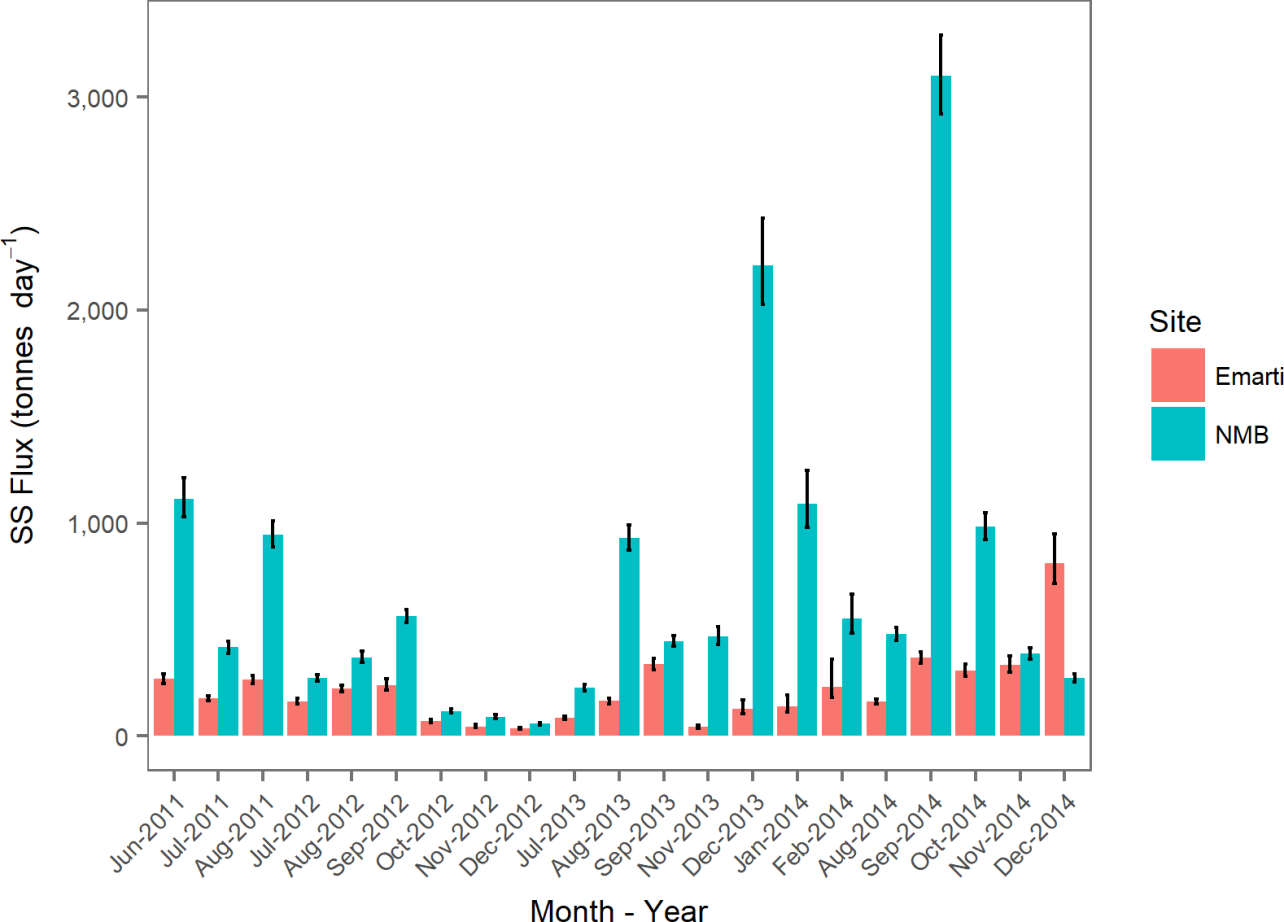
Mean monthly suspended sediment flux (tonne day^-1^) for Emarti and NMB sites. 95% confidence intervals are provided.

Turbidity ranged from 28 NTU to over 4000 NTU at Emarti and from 338 NTU to over 4000 NTU at NMB (Fig 6). Daily mean sediment fluxes from Emarti ranged from 35 tonnes day^-1^ (_- 10%_^+ 12%^) in December 2012 to 812 tonnes day^-1^ (_- 12%_^+ 17%^) in December 2014 (Fig 7). Daily mean sediment fluxes from NMB ranged from 57 tonnes day^-1^ (_- 9%_^+ 11%^) in December 2012 to 3100 tonnes day^-1^ (_- 6%_^+ 6%^) in September 2014. Flux was higher at Emarti than NMB during one month, indicating the potential for storage of sediments within the river channel between Emarti and NMB. Over the entire length of record, average suspended sediment flux at Emarti was 220 tonnes day^-1^ (_-11%_^+ 20%^) and at NMB it was 710 tonnes day^-1^ (_-7%_^+9%^). Sediment yield was similar for all three regions (Table 3).

**Table 3:** Sediment yields.

Dates	Region	Watershed Area (km^2^)	Yield (tonnes km^-2^ year^-1^)	95% Confidence Intervals	
				Lower	Upper
2011–2014	Emarti	2450	33	- 11%	+ 20%
	Middle Mara + Talek	4050	44	- 13%	+ 22%
	NMB (Emarti + Middle Mara + Talek)	6500	40	- 7%	+ 9%

### Sediment dynamics of hippo pools

We found that turbidity was significantly higher downstream at 3 of the 4 hippo pools we studied (Table 4 and S2–S5 Figs). Upstream and downstream turbidity measurements were statistically different at Amani Mara, Serena and OMB, but not statistically different at Ngerende. Variance in turbidity was significantly higher downstream of all hippo pools as was the coefficient of variation for turbidity.

**Table 4:** Hippo pool sampling summary statistics.

Hippo Pool		No. turbidity measurements	Turbidity (NTU)							
			Mean (NTU)	Standard Deviation	V—statistic	p—value	Variance	F—value	p—value	Coefficient of Variation
Ngerende	Upstream	1479	81.8	17.9	549720	0.5759	323	5.86	0.016	22%
	Downstream	1479	83.7	24.6			608			29%
OMB	Upstream	1411	280	17	452760	0.007	287	13.6	< 0.001	6%
	Downstream	1411	291	171			29246			59%
Serena	Upstream	2838	225.6	28.5	1312900	< 0.001	813	100	< 0.001	13%
	Downstream	2838	224.9	132			17424			59%
Amani	Upstream	1217	1524	135	700700	< 0.001	18312	105	< 0.001	9%
	Downstream	1217	1694	220			48519			13%

## Discussion

### Hydrology

During baseflow conditions, discharge is often higher at the Emarti site, with an estimated loss of approximately 1 m^3^ s^-1^ from Emarti to NMB [53]. Discharge was never below 3 m^3^ s^-1^ at Emarti. However, discharge was below 3 m^3^ s^-1^ at NMB during 5 separate months. Flow essentially stopped in the Mara River at the NMB site in September 2012, while mean discharge at Emarti was approximately 13.8 m^3^ s^-1^. This is likely indicative of over-abstraction in the Middle Mara region, although natural factors such as evaporation and loss to groundwater likely contribute as well. Most of the tourism lodges on the river (downstream of Emarti) report abstracting water directly from the river for their use, and their water consumption increases during the high season of July through October, when the wildebeest migration is present in this region [54]. Abstraction during the dry season by hoteliers may be significant, but there are no available records for abstraction rates in the region. Wildlife consumption of water also has been speculated to impact the flows in this area [53].

NMB had a consistently higher R-B Index, indicative of flashier discharge at this site. We would expect NMB to have a lower R-B Index because it drains a catchment approximately 3 times the size of Emarti so flows should be more stable. In spite of this, the NMB site is in the highest quartile of the R-B Index compared to rivers of a similar drainage while the Emarti site is in the lowest quartile [48]. The R-B Index increased each year at NMB, although this increase could be a result of sampling during drier months in the first year followed by the sampling of progressively wetter months in the years that followed. The R-B Index has lower inter-annual variability and greater power to detect trends when compared to other flow indices, and it provides a useful index for comparison to other sites or rivers [48]. The R-B Index values calculated for this study to compare Emarti and NMB are robust because only data that was available for both sites for the same time periods was used to create the index. However, we caution against using them as a direct comparison to other rivers since we used data that did not include the entire annual cycle. The discharge values reported here are similar to those for the Mara in the historical record. Site 3 of the Mara River Environmental Flows Assessment (EFA), conducted between 2006–2010, is within 200 meters of the NMB site from this study [27]. During the EFA study, discharge at the NMB site was calculated by applying a watershed transformation factor to discharge data from a monitored site several kilometers downstream, Mara Mines. The flow duration curve created for Site 3 as part of the EFA indicates that discharge at the NMB site exceed 11 m^3^ s^-1^, 50% of the time within each year [27]. Our calculated average at NMB over the four years of sampling was very similar at 12.5 m^3^ s^-1^ (_- 15%_^+ 19%^). Likewise, a recent study of the historical records for the Amala and Nyangores rivers reported an annual combined mean discharge of 12.45 m^3^ s^-1^ [45]. The mean discharge for this study at the Emarti site (7 km downstream of the confluence of the Amala and Nyangores rivers) is similar to this sum with an average discharge of 13.3 m^3^ s^-1^ (_- 23%_^+ 32%^).

Periods of data collection with overlap were not available for the typical peak wet season (April-May). The absence of these peak discharge periods in our data could have led to an underestimation of total sediment flux and yield from each portion of the watershed. Additionally, the absence of data in several low flow months (January and February) could lead to an overestimation of the total sediment flux and yield. However, we did capture several seasons of data from the other high and low discharge months for Emarti and NMB, and comparison of our overall discharge data to several other studies gives support to our conclusions.

### Sediment fluxes

The suspended sediment flux at Emarti is approximately 1/3 of that at NMB. The bulk of the sediment flux appears to be generated in the semi-arid Middle Mara and Talek regions. Since Emarti to NMB is a hydrologically losing reach, our flux calculations may overestimate the contribution of the Middle Mara and Talek regions if previously sequestered in-channel sediments from Upper Mara are remobilized from within the channel during floods. However, previous research during a three-month period in 2011 using a sediment fingerprinting method has supported the finding that 2/3 of the suspended sediments at NMB are coming from the Middle Mara and Talek regions and are not the result of the remobilization of in-channel sediments from the Upper Mara [35]. Furthermore, this fingerprinting study showed that 50% of the suspended sediment flux at NMB came specifically from the Talek region. [35].

Sediment flux at NMB was on average over 253,000 tonnes year^-1^, and sediment flux at Emarti was only 81,000 tonnes year^-1^. Average monthly fluxes were more variable at NMB (higher CV) with an increase as high as 600% between August and September, 2014, due to a marked increase in discharge. Monthly fluxes were much more stable at Emarti. The largest increase in monthly flux at Emarti was approximately 300% over the previous month. Interestingly, fluxes at Emarti dropped 800% between September and November, 2013. This was likely a consequence of the reduction of mean flow from 17 m^3^ s^-1^ to 7 m^3^ s^-1^ between September and November.

We found that the sediment yields across the three catchments were not significantly different from one another. It is not possible to separate the Middle Mara and Talek regions in the yield calculations, although previous analyses using a sediment fingerprinting approach have suggested that the Talek has a higher yield than the Middle Mara region [35]. The Middle Mara is within the MMNR, a conservation area with strict development rules, while the Talek catchment is a mix of pastoralists, wildlife conservancies and tourism developments.

Our findings are contrary to the traditional model of mountain streams having a disproportionately large influence on the sediment yield of a river due to high levels of precipitation and elevation change [55]. Although mountain streams drain an estimated 20% of the total land area on Earth, they provide up to 50% of the sediment supply to the oceans [56]. The Upper Mara is half the size of the semi-arid Middle Mara—Talek catchment but receives twice as much rainfall; thus, we expected the Upper Mara to have a higher sediment yield. However, we did not find a significant difference in yield between the two. The higher than expected sediment yields from the Middle Mara–Talek catchment may be due to grazing by livestock in this region. An earlier study on sediment yields in Kenya found that grazing lands provided the highest sediment yield [57], and increased erosion in the Loita Hills (which are in the Talek catchment) has previously been attributed to cattle [58]. Glover et al. (1958) found that cattle increase erosion more than wildlife due to their herding behavior and tendency to stay in one area longer than wildlife. The shearing action of hooves breaks through the armored soil layer leading to an increase of finer particles on the surface and an increase in erosion [59]. Rapp (1977) also found that the most important erosion process occurring in a semi-arid catchment within Tanzania was sheetwash from grazing and unprotected cultivations [60]. Vegetation cover and land use are important factors controlling overland flow-induced erosion from landscapes regardless of the underlying lithology [61]. Overgrazing has been cited as one of the principal mechanisms contributing to the desertification of rangelands [62].

The Talek region appears to have experienced landscape degradation as early as the 1970s. Between 1980 and 2000, there was a 70% decrease in wildlife on the Mara Rangelands that was attributed in part to the conversion to wheat cultivation in the Loita Plains, which are situated partly within the Talek watershed [63, 64]. Recent studies have found that most wildlife species have declined by more than 2/3 between 1977 and 2009 in the greater Mara Ecosystem, and domestic animal populations have increased rapidly [65]. Mechanized farming has expanded from 4,800 ha to 47,000 ha around the Maasai Mara National Reserve between 1975 and 1995 with most of the changes occurring in the Loita Plains [63, 66]. With continued population expansion, land use changes, and climatic variability, it is likely that sediment loading will continue to increase from the Talek region. Accurately quantifying the soil erosion caused by livestock in the Talek catchment remains an important research priority.

Suspended sediment loads are highly episodic. Up to 90% of the annual mass of sediments can be transported within just 10% of the time [67]. Sub-daily measurements are essential for accurately calculating flux and yield for a flashy river [14]. We used sub-hourly discharge and turbidity data, combined with a turbidity-suspended sediment relationship, to generate our flux and yield calculations instead of developing a relationship directly between discharge and suspended sediment concentration. Developing a direct relationship between discharge and suspended sediment concentration can result in very large errors if used in the estimation of suspended sediment fluxes and yields because that relationship can vary across time [68].

Although we captured a range of hydrological events from all portions of the study area, it is possible that we did not catch sediment transport that would be representative of a full hydrological year. Several months of low flow and high flow were missed each year due to sonde failure and logistical constraints. Our measurements are likely an underestimate of the true flux and yield, as the measurement range on the turbidity sensor was exceeded multiple times. For calculation purposes, SSC, flux and yield were made with the maximum value, which would be an under-representation of the true value during those extreme events.

When calculating flux and yield estimates, the propagation of uncertainty throughout each measured value provided cumulative error estimates in the range of 5% to 55%. The largest confidence intervals were for low flux values for the NMB site and high flux values for the Emarti site. Errors inherent in the discharge measurements and in the relationship between stage height and discharge were the primary source of uncertainty for sediment flux and yield estimates. Care had to be taken during collection of discharge measurements due to the high tourism environment, presence of large wildlife (hippos and Nile crocodiles) and the geomorphology of the river. We believe that the sacrifices in accuracy that we made were necessary to the overall success of gathering data in this type of environment.

### The role of hippos

In three out of the four hippo pools, there was an increase in mean turbidity downstream of hippo pools compared to upstream. There was also a large increase in the variance and coefficient of variation in turbidity downstream of all the hippo pools. Hippos likely increase river bed and bank erosion through wallowing behavior within the river and through creation of deeply incised paths through the riparian zone. Hippos also continually deposit significant amounts of new organic material along the bottom of the pool through defecation [39]. We have previously found that hippo feces is detectable utilizing a sediment fingerprinting approach and that it contributes up to 5% of the suspended sediments at NMB during baseflow [35]. These suspended sediments, along with others from upstream, may be trapped within the hippo pools due to lower current velocities, but they ultimately are mobilized downstream by hippo movements or scouring flows. This was particularly evident in the Amani hippo pool sampling event, as a rain event during sampling at approximately 1800hrs mobilized sediments and organic matter that were stored within the pool and created a marked increase in turbidity downstream compared to upstream (see S5 Fig). The influence of deposition and resuspension dynamics on sediments in hippo pools warrants further attention as they are likely important but were not addressed in this study.

## Conclusions

Our research shows that the highest sediment flux into the Mara River comes from the Middle Mara and Talek regions of the basin. Our findings are contrary to the generally accepted theory that deforestation in the Upper Mara is currently responsible for most of the suspended sediment loading in the river. The deforestation in the Mau Forest has likely impacted the hydrologic cycle of the Mara River, resulting in lower baseflows and higher peak flows, and also has likely increased sediment flux from that region [18, 19]. However, the protected areas and adjoining group ranches in the Middle Mara—Talek catchment are home to a resident population of several hundred thousand wild and domestic animals, as well as an additional 1.3 million herbivores every year during the wildebeest migration, and the presence of these animals and associated land use changes in this region have also significantly impacted sediment dynamics in the river [65]. Although we recognize the importance of conserving the Mara River headwaters to protect the river’s natural hydrological and sediment regime, we also suggest increasing attention should be given to conservation of the Middle Mara and Talek regions.

Our study also suggests that hippos may have complex effects on suspended sediment dynamics in rivers, with hippo pools acting as both sources and sinks for organic material and suspended sediments. Defecation and physical movements of hippos in pools may increase sediment loading and downstream transport, albeit in short pulses. However, hippo pools also reduce water velocity and increase sediment deposition. The net effect of hippo pools on sediment dynamics in rivers is largely influenced by discharge.

The drivers of sedimentation rates in the Mara River are an issue of tremendous political and conservation interest. The river is shared by two countries, and it is at the headwaters of the Nile River. In addition to sustaining some of the most famous protected areas in the world, it also provides domestic water sources for over half a million rural poor people. Concerns about water quantity and quality in the Mara have been a long-standing issue in the region, with potential consequences for the region’s economy, wildlife and human health. Furthermore, several dams are potentially planned for the Mara River, which could significantly alter sediment regimes in the basin [69]. Our research elucidates several previously unexplored drivers of sediment dynamics in this important river system and demonstrates that a full understanding of sediment dynamics in the Mara, and perhaps other sub-Saharan African rivers, cannot be achieved without considering the role of semi-arid catchments, livestock grazing and large wildlife.

## Supporting information

S1 Fig Stage height to discharge rating curves for June 2011 –Nov 2012 at NMB extrapolated over the full range of measured stage height. Outliers that were removed are presented as a triangle. 95% confidence intervals are in red.
(TIF)

S2 Fig Upstream and downstream turbidity measurements taken every minute over a 24-hour period in the Ngerende hippo pool. (TIF)

S3 Fig Upstream and downstream turbidity measurements taken every minute over a 24-hour period in the OMB hippo pool. (TIF)

S4 Fig Upstream and downstream measurements of turbidity taken every minute over a 48-hour period in the Serena hippo pool. (TIF)

S5 Fig Upstream and downstream turbidity measurements taken every minute over a 24-hour period in the Amani hippo pool. (TIF)

S1 Table Time blocks created with the number of measurements within each time block. (XLSX)

S2 Table Percent monthly coverage and number of measurements per month and year. (XLSX)

S3 Table Hippo pool monitoring offset and correlations. (XLSX)
