# Supplementary figures and images for: The influence of a semi-arid sub-catchment on suspended sediments in the Mara River, Kenya
Source: PLoS One. 2018 Feb 8;13(2):e0192828. doi: 10.1371/journal.pone.0192828 (PMC5805331; doi:10.1371/journal.pone.0192828)

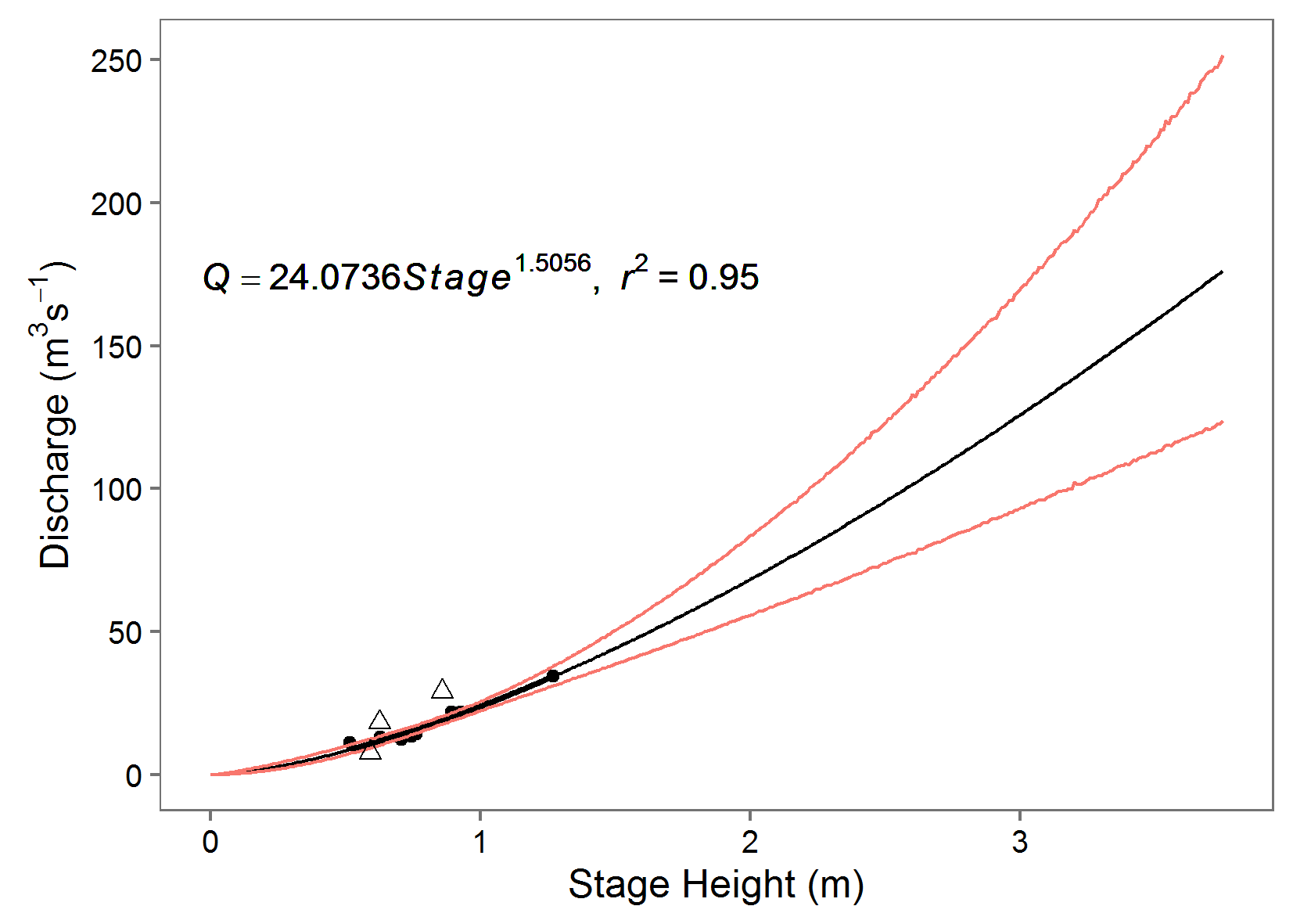

Supplement: S1 Fig — Outliers that were removed are presented as a triangle. 95% confidence intervals are in red. (TIF) [file pone.0192828.s001.tif]

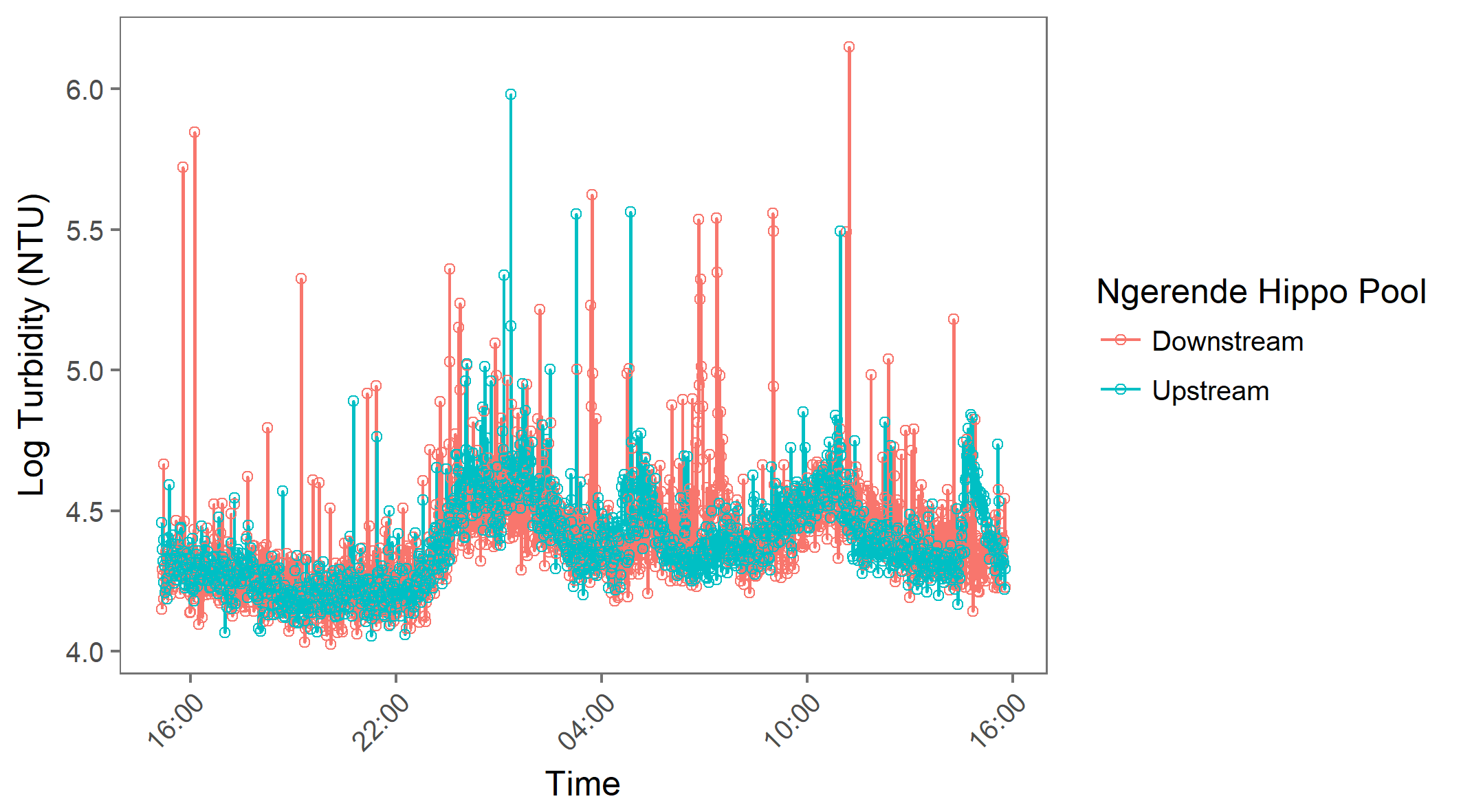

Supplement: S2 Fig — (TIF) [file pone.0192828.s002.tif]

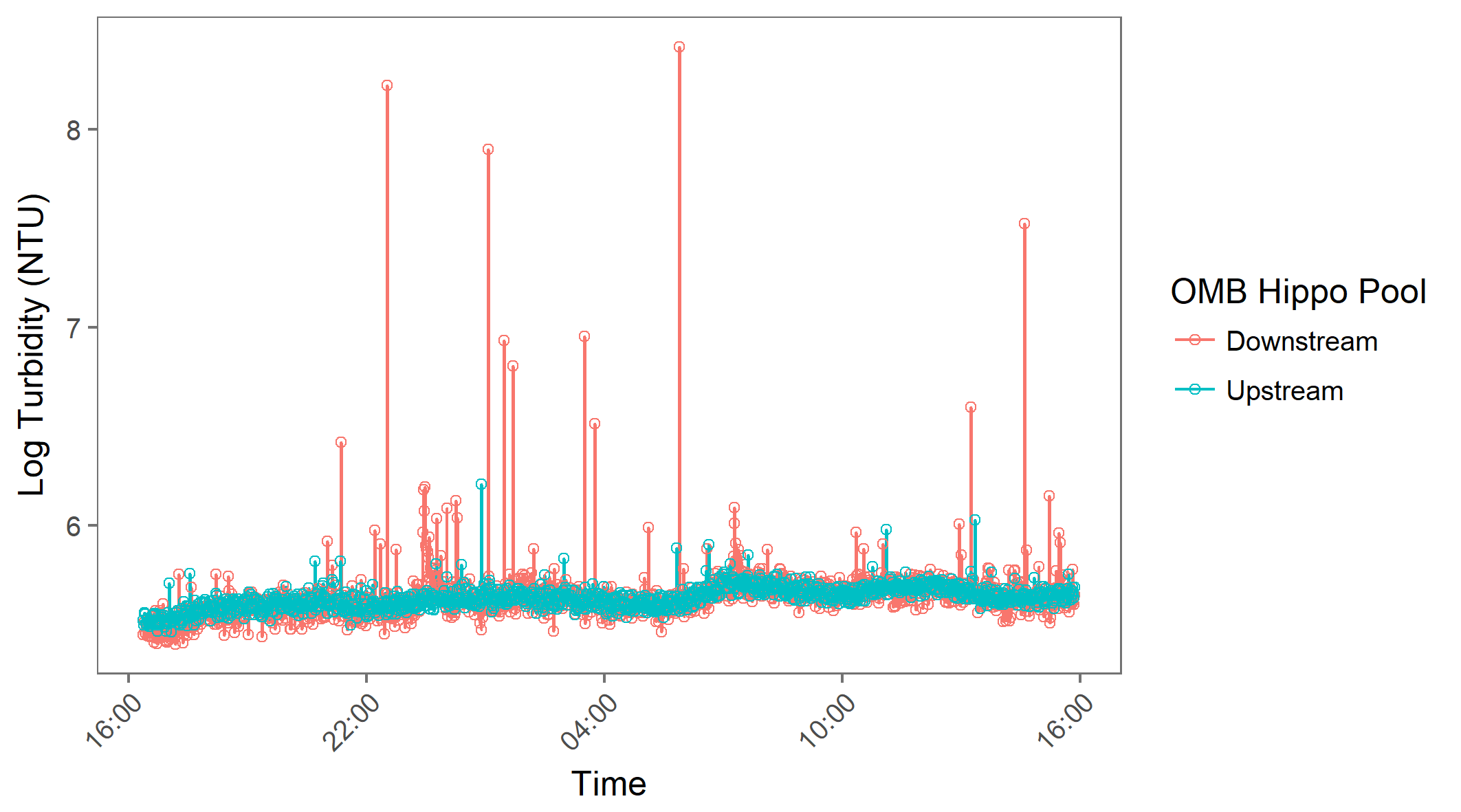

Supplement: S3 Fig — (TIF) [file pone.0192828.s003.tif]

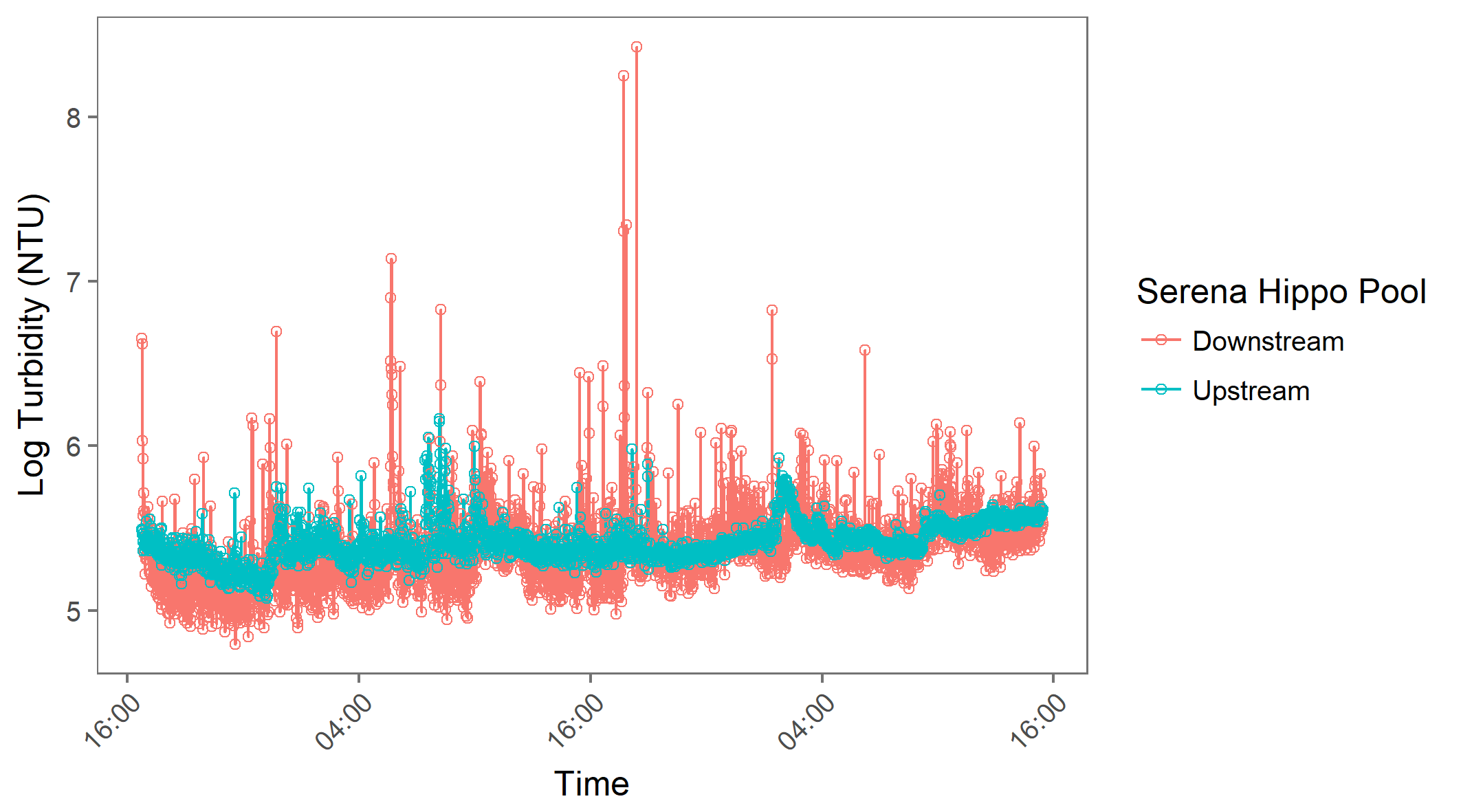

Supplement: S4 Fig — (TIF) [file pone.0192828.s004.tif]

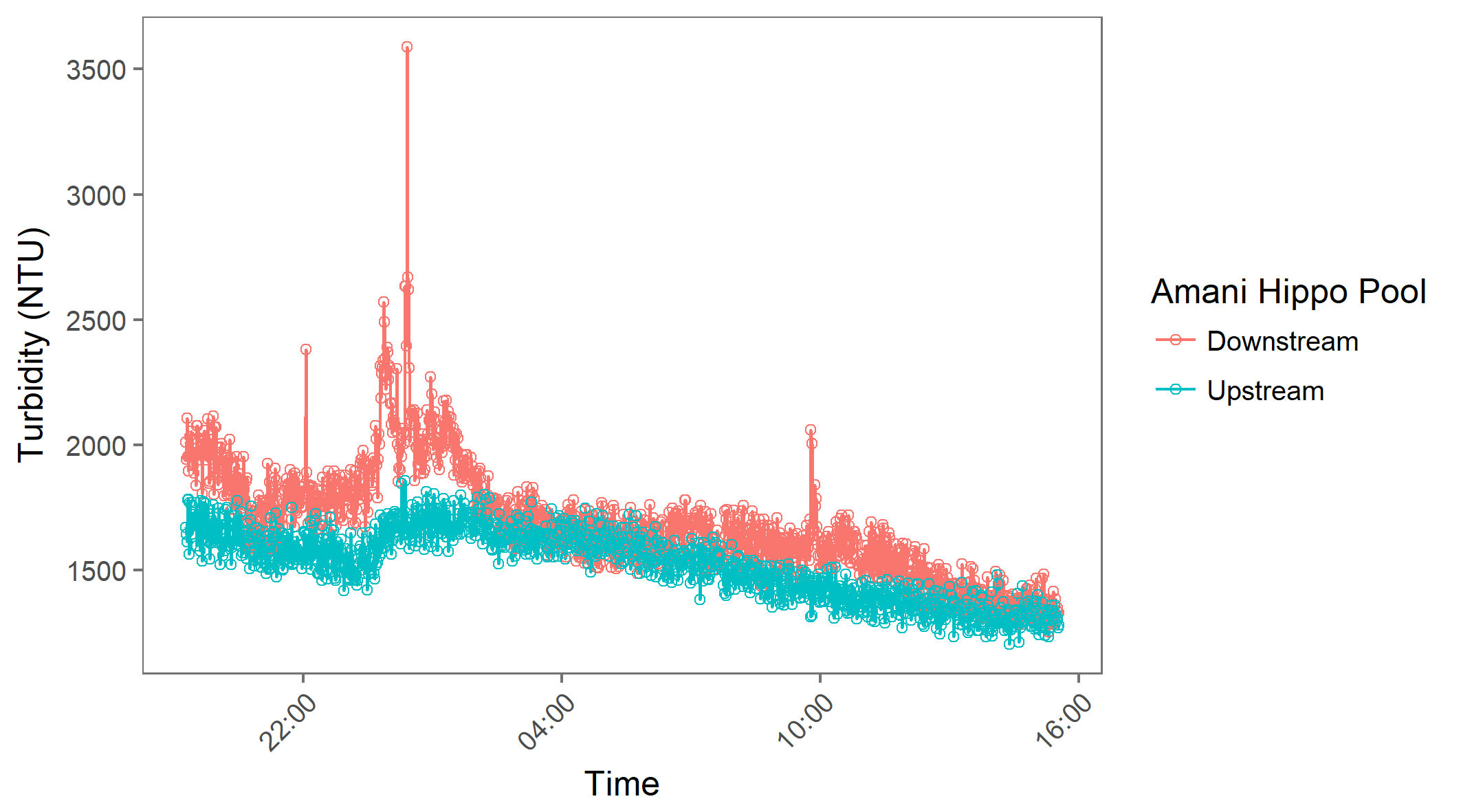

Supplement: S5 Fig — (TIF) [file pone.0192828.s005.tif]
